# Porcine to Human Heart Transplantation: Is Clinical Application Now Appropriate?

**DOI:** 10.1155/2017/2534653

**Published:** 2017-11-07

**Authors:** Christopher G. A. McGregor, Guerard W. Byrne

**Affiliations:** ^1^Institute of Cardiovascular Science, University College London, London, UK; ^2^Department of Surgery, Mayo Clinic, Rochester, MN, USA

## Abstract

Cardiac xenotransplantation (CXTx) is a promising solution to the chronic shortage of donor hearts. Recent advancements in immune suppression have greatly improved the survival of heterotopic CXTx, now extended beyond 2 years, and life-supporting kidney XTx. Advances in donor genetic modification (B4GALNT2 and CMAH mutations) with proven Gal-deficient donors expressing human complement regulatory protein(s) have also accelerated, reducing donor pig organ antigenicity. These advances can now be combined and tested in life-supporting orthotopic preclinical studies in nonhuman primates and immunologically appropriate models confirming their efficacy and safety for a clinical CXTx program. Preclinical studies should also allow for organ rejection to develop xenospecific assays and therapies to reverse rejection. The complexity of future clinical CXTx presents a substantial and unique set of regulatory challenges which must be addressed to avoid delay; however, dependent on these prospective life-supporting preclinical studies in NHPs, it appears that the scientific path forward is well defined and the era of clinical CXTx is approaching.

## 1. Introduction

About 5.7 million Americans have heart failure, half of whom will die within 5 years [1]. Organ transplantation is currently the preferred solution for treatment of end-stage heart failure but less than 3000 heart transplants have been performed annually in the US in recent years. Circulatory assist devices and total artificial hearts have been approved to support patients in chronic heart failure [2, 3]. These mechanical solutions are effective, at least in the short term, but have significant morbidity from thromboembolism, infection, gastrointestinal bleeding, and reduced quality of life [4]. Regenerative solutions for heart failure remain a nascent experimental technology. Cardiac xenotransplantation (CXTx) is a promising viable near-term solution to the shortage of hearts for clinical transplantation. In recent years, there has been a remarkable improvement in survival of heterotopic pig-to-nonhuman primate (NHP) CXTx [5–8], encouraging early success in orthotopic CXTx (oCXTx) [9–11] and advances in life-supporting renal xenotransplantation (RXTx) [12, 13]. These results validate the physiological compatibility of porcine organs, at least in NHPs, and suggest that clinical CXTx may soon be applicable if oCXTx can attain similar improvements in survival as RXTx. In this review, we examine developments in immune suppression, porcine donor genetics, preclinical transplants, and infectious disease issues and discuss requirements for clinical CXTx.

To justify a clinical xenotransplantation (XTx) program, it is necessary to demonstrate transplant efficacy in clinically relevant animal models. The International Society of Heart Lung Transplantation (ISHLT) has suggested that a prospectively defined series of life-supporting cardiac xenotransplants in NHPs, using predefined immune suppression, with “60% survival at 3 months with a minimum of 10 animals surviving for this period,” would be sufficient to consider a clinical trial [14]. The recent survival achieved after heterotopic CXTx (hCXTx), in excess of 2 years and with a median survival of 298 days [8], suggests that this goal may be attainable. While the ISHLT recommendation has become a de facto guideline for researchers in the field, the FDA, responsible for regulating XTx, has not officially endorsed these specific criteria, making early interactions with regulators essential to advance a clinical study.

The most prominent variables which contribute to XTx efficacy are likely to be immune suppression and donor genetics, but recipient species [15], viral status [16–18], the level of preexisting anti-pig antibody [13], prophylactic antiviral and antibacterial therapy [19, 20], and postoperative care [21] also significantly contribute to graft survival. The relative contribution of immune suppression and donor genetics is incompletely understood in a field undergoing rapidly evolving experimental changes in both components. Past reviews of the state-of-the art in XTx [22–24] and recent publications [8, 25] are useful for gaining an appreciation for the breadth of changes in XTx organ survival over the last 20 years.  Table 1, showing the longest reported (and median) graft survival for various donor genetics under four broadly defined immune suppression regimens, summarizes these advances for CXTx. Not surprisingly, all combinations of donor genetics and immune suppression have not been reported, as perceived advances in donor genetics were seldom tested with reduced or previous immune suppression techniques.

## 2. Earlier Immune Suppression

Early hCXTx studies using Gal-positive (WT) donor hearts focused on preventing hyperacute rejection and often used immune suppression based largely on cyclophosphamide, cyclosporine, and steroids (CCS) [26]. Maximal hCXTx survival of 32 days was achieved in pig-to-cynomolgus monkey transplants using soluble CR1 to block systemic complement activation [27]. Comparable results were also reported after pig-to-baboon hCXTx using cobra venom factor (CVF) to consume complement [28]. CCS immune suppression was also used in early hCXTx studies using transgenic donors expressing human complement regulatory proteins (hCRPs). The longest reported survival was 99 days (median 26 days) using hDAF transgenic hearts [29]. These results demonstrated that expression of hCRPs was sufficient to abrogate the need for systemic complement inhibition, but was not sufficient to prevent an induced antibody response and antibody-mediated rejection (AMR). When anti-Gal antibody was blocked *in vivo* using a Gal polymer, more consistent graft survival, median 35 days, was reported in pig-to-cynomolgus monkey hCXTx [30]. This study appears to be the first to detect an induced non-Gal antibody associated with xenograft rejection. These early CCS regimens were often poorly tolerated due to the narrow therapeutic index for cyclophosphamide.

An alternative immune suppression strategy based on induction with ATG and Rituximab and using tacrolimus and sirolimus maintenance immune suppression was used in WT;hCRP, GGTA-1 *α*-galactosyltransferase-deficient pigs (GTKO), and GTKO;hCRP transplants. The studies with WT;hCRP donors involved the largest series of transplants (*n* = 63) using a Gal polymer to test the effects of systemic anticoagulation and immune suppression on graft survival [31]. At moderate tacrolimus and sirolimus maintenance levels, hCXTx graft survival of up to 109 days (median survival 20 days) was achieved, similar to earlier results using CCS immune suppression. At higher levels of maintenance immune suppression [32, 33], maximal survival was improved (139 days) with more consistent and prolonged median survival of 96 days. This was the first instance of median cardiac xenograft survival in excess of 3 months. In these WT;hCRP studies, anti-Gal-mediated rejection was minimized and graft rejection was associated with non-Gal antibody. Moreover, testing three distinct, tightly controlled clinical anticoagulation therapies did not improve graft survival or affect the histology of graft rejection, indicating no strict requirement for, or benefit from, systemic anticoagulation. Using GTKO or GTKO;hCRP donors with moderate tacrolimus and sirolimus, immune suppression achieved comparable survival to earlier WT;hCRP transplants using Gal polymers, indicating that the adoption of GTKO donors obviated the need for specific therapy to control anti-Gal antibody and suggested that graft survival was limited by the effects of non-Gal antibody [34]. Also, the three-month median survival achieved in these hCXTx studies made the conversion to oCXTx the appropriate model of choice moving forward.

## 3. Costimulation Blockade Immune Suppression

The more recent form of immune suppression utilized in hCXTx is costimulation blockade, primarily directed at the CD154 (CD40 ligand) and CD40 secondary signaling pathway, to block Th2 cell help for B cell activation. Antibody to CD154, originally shown to suppress allograft rejection [35], has been used extensively in pig-to-NHP hCXTx with donor organs ranging from WT;hCRP to GTKO;hCRP donors (Table 1). This immune suppression regimen was complex, including lymphocyte depletion with ATG and LoCD2b antibody, complement inhibition with CVF and steroids, and chronic postoperative immune suppression with mycophenolate mofetil and anti-CD154 antibody (5c8), and usually supplemented with a range of anticoagulant therapies. Early versions of this protocol also included pretransplant thymic irradiation [36] and used Gal polymers. With WT;hCRP donor hearts, maximal hCXTx organ survival was 139 days with a median of 23 days. More consistent organ survival was achieved (median 78 days) by transplantation of GTKO donor hearts into recipient baboons with little or no detected preformed non-Gal antibody [37]. In both instances, there was no apparent induction of circulating Gal or non-Gal antibody although the histology of the graft at explant, showing vascular antibody and complement deposition, was consistent with AMR. Importantly, anti-CD154 costimulation blocking regimens often reported complications with thrombocytopenia, consumptive coagulopathy (CC), and systemic inflammation which contributed to recipient loss [38, 39]. With GTKO;CD46 donor hearts, 236-day maximal graft survival (median survival 71 days) was achieved using a modified anti-CD154 protocol which included Rituximab induction to deplete B-cells [5]. Intensive postoperative monitoring in this study also likely contributed to prolonging graft survival. Explanted grafts showed evidence of ongoing humoral rejection; however, the authors indicated that survival was largely limited by nonimmune model-related issues for managing the recipient animals and recurrent thrombocytopenia, ascribed to the use of anti-CD154. The continued use of CVF, even with GTKO;CD46 donor organs, also contributes to systemic coagulation perturbations [40], precluding its clinical use.

Systemic thrombocytopenia and CC associated with anti-CD154 binding to activated platelets are well known, and significant efforts have been made in XTx models to find an effective substitute costimulation blockade regimen [41, 42]. Recent studies demonstrate that chronic administration of an anti-CD40 blocking antibody (2C10R4), substituting for anti-CD154, leads to prolonged hCXTx survival [25]. In the initial report, anti-CD40 (2C10R4), administered for just 60 days posttransplant, achieved maximal GTKO;hCRP hCXTx survival of 149 days and median survival of 84 days [25]. Graft survival appeared to be limited by the dosage and duration of immune suppression as withdrawal of anti-CD40 therapy resulted in a marked rise in antibody titer and xenograft rejection. Importantly, substituting anti-CD40 antibody for anti-CD154 moderated complications of thrombocytopenia and consumptive coagulopathy, which may also have contributed to improved graft survival. Using a higher dosage of anti-CD40 (2C10R4), administered for longer, resulted in longer survival of GTKO;hCRP donor hearts expressing human thrombomodulin (TBM) with maximal survival of 945 days (median survival 298 days) [8]. These outcomes likely underestimate survival as anti-CD40 therapy was reduced in 2 of 5 recipients after 100 days and was reduced for two other recipients after 1 year. In these latter recipients, anti-CD40 therapy was eventually withdrawn at 560 and 861 days posttransplant. In each case, reduction/cessation of anti-CD40 therapy resulted in induction of non-Gal IgM and IgG antibody with eventual graft rejection. Despite persistent vascular expression of human TBM, the histology of explanted rejected hearts exhibited features typical of xenograft rejection including thrombotic microangiopathy, vasculitis, intravascular thrombosis, and myocardial necrosis with little evidence of lymphocytic infiltration suggesting limited impact of the TBM addition. Recipients also received CVF, heparin, and aspirin.

The new anti-CD40-based costimulation blocking regimen appears to have achieved a level of humoral and cellular immune suppression which, for the duration that it is provided, blocks non-Gal AMR, with graft survival now measured in years. This appears to surmount a major obstacle to clinical XTx. While the current anti-CD40 (2C10R4) is a mouse/rhesus chimeric IgG4 antibody which would not be suitable for use in humans, it is reportedly being humanized [6]. There are also several other humanized anti-CD40 blocking antibodies under various levels of development (Table 2), suggesting there may soon be a clear path forward for immune suppression, using only approved therapies, to support clinical CXTx.

## 4. Donor Genetics

Genetic engineering of the donor pig is a cornerstone of XTx as it enhances organ survival and function, while reducing the need for systemic therapies in the recipient. The pace of genetic manipulation of the pig genome has significantly increased with the introduction of somatic cell nuclear transfer and sequence-directed nucleases [43, 44]. This proliferation currently outstrips the pace of analysis in pig-to-NHP transplants. There are now dozens of reported gene additions or deletions [45, 46] with suggestions that donor animals with 5 or more genetic alterations affecting complement regulation, antigen reduction, haemostatic incompatibilities, coagulation dysfunction, suppression of inflammation, adaptive T-cell immunity, and endogenous retrovirus infectious risks may be required for clinical XTx [47]. In the search for an “ideal” donor, the simultaneous introduction of multiple genetic modifications, without appropriately controlled experiments, may obfuscate their function, as well as introduce unnecessary complications. A consistent strategic approach to developing and testing new donor genetics would accelerate the application of clinical CXTx. Moreover, the accumulation of multiple gene modifications complicates donor breeding programs to the point that somatic cell cloning may be required to maintain the genetic profile. This will increase cost and may limit the production of donor animals for preclinical studies, the results of which have already been frequently compromised by small group sizes.

Donor genetic modifications have focused on four main categories, antigen reduction [48], thromboregulation [49], immune suppression, and infectious disease [50]. Two additional non-Gal glycan antigens have been identified, N-glycolylneuraminic acid- (Neu5Gc-) modified oligosaccharides [51, 52] and the glycan product synthesized by porcine beta 1,4-N-acetylgalactosamine transferase-2 (B4GALNT2) [53]. Humans do not synthesize Neu5Gc due to a mutation in the CMP-N-acetylneuraminic acid hydroxylase gene (CMAH), but they do produce an array of antibodies which show Neu5Gc-dependent reactivity to sialylated oligosaccharides. These anti-Neu5Gc antibodies are noted for their role in serum sickness in patients treated with animal sera [54]. The B4GALNT2 gene catalyses the terminal addition of N-acetylgalactosamine (GalNAc) to a sialic acid-modified lactosamine acceptor producing GalNAc*β*4[Neu5Ac*α*2,3]Gal *β*4GlcNAc*β*3Gal, the SDa blood group antigen. This is an immunogenic glycan in pig-to-NHP CXTx [55]. Humans are known to produce low levels of IgM which bind the polyagglutinable human SDa blood group [56–59]. Targeted mutations affecting the porcine CMAH and B4GALNT2 genes have been made and combined with the GTKO mutation [48, 60–62]. These three mutations together minimized human IgM and IgG binding to porcine cells in over 90% of human serum samples [48] and reduced both IgM and IgG reactivity to background levels in 30% of allosensitized wait-listed renal transplant candidates [60]. Pig-to-NHP CXTx using organs with these mutations has not yet been reported. Minimizing tissue immunogenicity would appear to have obvious clinical benefit but demonstrating this in preclinical transplants will be difficult as NHPs do not produce anti-Neu5Gc antibody. Additionally, induction therapy with ATG, part of the current costimulation blocking immune suppression, may induce an anti-Neu5Gc antibody response in humans [63], which is not apparent in NHPs. This induced response could sensitize recipients and compromise Neu5Gc-positive donor organs in clinical CXTx. This suggests that new large animal transplant models, using CMAH-deficient recipients, may be required to test the pathogenicity of anti-Neu5Gc antibody and optimize the use of biological agents for immune suppression. Despite these issues, genetic engineering directed at reducing the antigenicity of porcine tissue is likely to significantly impact clinical CXTx.

Interest in transgenic augmentation of thromboregulation stems primarily from recognition of molecular incompatibilities between porcine and human TBM [64]. Secondarily, immune-independent recipient and donor cell-to-cell interactions have been described *in vitro* which are proposed to contribute to donor endothelial cell activation [65, 66], systemic haemostatic dysfunction, and CC [67–69]. Several groups have reported production of human TBM transgenic pigs [70–72], and *in vitro* analysis of porcine endothelial cells expressing human TBM shows that it alleviates the molecular incompatibility with efficient production of activated human protein C [64, 73]. A limited number of pig-to-NHP CXTx studies with human TBM expressing donor organs have been reported [8, 74]. The impact of human TBM expression cannot be determined from these studies as their designs lacked controls without TBM, included chronic systemic heparin administration, and had no direct measure of human TBM function. Transgenic expression of other key components which affect haemostasis, CD39, CD47, TFPI, and EPCR, has also been reported [12, 75].

The common donor modifications, transgenic expression of hCRPs, and the GTKO mutation have been thoroughly analysed and validated. Physiological or supraphysiological vascular expression of one or more human complement regulatory genes, CD59, CD55, or CD46, establishes an intrinsic barrier which regulates the complement cascade [76–78], reduces the incidence of hyperacute rejection, and limits complement-mediated injury [15]. Likewise, the significance of anti-Gal antibody and benefit of targeted mutation of the porcine *α*-galactosyltransferase locus [79–81] have been extensively documented in *in vitro* [82] and *in vivo* studies in both pig-to-NHP [83–86] and GTKO mouse models [87–91]. The combination of hCRP and GTKO donor modifications has also been specifically examined and demonstrated to be beneficial, preventing rare hyperacute GTKO hCXTx rejection and early immune injury [34, 92]. This basic genetic background, GTKO;hCRP, represents, in our judgement, the current, proven starting base for any clinical study. Additional CMAH- and B4GALNT2-directed antigen reduction would appear to be a beneficial clinical priority.

## 5. Orthotopic CXTx and Perioperative Graft Function

The vast bulk of CXTx studies have to date utilized an abdominal non-life-supporting heterotopic transplant model where the graft is contractile but does not support the recipient's circulation. In comparison, there have been a limited number of oCXTx studies [9–11, 93–99]. These studies, predominantly using WT;hCRP donors, report healthy recipient survival up to 57 days [46]. In this difficult model, recipient death is often due to postoperative management limitations, with explanted hearts often showing little histologic evidence of significant rejection. These studies, which could not yet utilize the most recent highly successful costimulation blockade immune suppression, clearly indicate that porcine hearts can provide life-sustaining and adequate circulation to NHPs and suggest that the efficacy of oCXTx is not intrinsically limited by cardiac function but by immune rejection and postoperative management. To demonstrate life-supporting oCXTx in a preclinical NHP model, consistent with the ISHLT guidelines, will require not only effective immune suppression and appropriate donor genetics but also substantial clinical level resources, expertise, and postoperative management.

Orthotopic CXTx studies also unmasked a potential impediment to clinical CXTx which was not apparent in hCXTx studies. Every research group that has performed oCXTx has reported variable perioperative mortality ranging from 40 to 60% within the first 48 hours. Xenograft failure in this time period was not due to hyperacute rejection as the explanted hearts show vascular antibody deposition but otherwise normal myocardial histology [100]. Instead, early graft failure was associated with primary organ dysfunction. We have called this phenomenon perioperative cardiac xenograft dysfunction (PCXD) which at this high frequency currently represents a significant barrier to clinical CXTx. Our ongoing studies suggest that PCXD is similar to ischemia reperfusion injury or cardiac stunning. We find that PCXD can be modulated with a preconditioning regimen to reduce circulating antibody, B-cells, and plasma cells prior to transplant, coupled with improved organ preservation [101]. In recipients which survive beyond 48 hours, PCXD is less evident and echocardiographic analysis indicates that PCXD is completely reversible showing that the normal cardiac reparative processes function across the XTx barrier [11]. Intrathoracic heterotopic cardiac transplantation, where both the donor and recipient hearts contribute to the circulation, was successfully used in early allotransplantation when techniques for donor organ preservation were being optimized. Preclinical intrathoracic heterotopic cardiac xenotransplantation studies, although complex, potentially offer a unique opportunity to study the aetiology and recovery from PCXD [102, 103]. Genetic engineering approaches may also have the potential to mitigate PCXD, for example, by providing high levels of CD39 expression [75] or reducing sodium hydrogen ion exchange activity [104].

## 6. Diagnosis and Treatment of Rejection

The ability to diagnose and treat rejection is a key component of clinical transplantation. The ISHLT has developed pathologic grades (pAMR1–3) of immunopathologic features of endomyocardial biopsies which along with graft dysfunction and levels of donor-specific antibody are used for the diagnosis AMR in cardiac allotransplantation [105]. The most severe pathology (pAMR3), associated with significant graft dysfunction and poor clinical outcomes, can be treated with a combination of increased and optimized immune suppression, depletion of circulating antibody, and IVIg. More aggressive salvage therapies may also include B-cell and plasma cell depletion and complement inhibition [105, 106]. In CXTx, there are few studies which have attempted to diagnose and treat presumptive rejection episodes, most of these after hCXTx [9, 25, 33, 107–109]. Putative rejection episodes were diagnosed based on biochemical markers (troponin T, AST), graft contractility, telemetric measures of cardiac performance, and echocardiography. Serial biopsies after oCXTx will likely be applicable for diagnosis of rejection [109–111], but the difficulty of obtaining endomyocardial biopsies in NHPs has limited their exploration in animal models. When presumptive rejection episodes were treated using steroids, or steroids and ATG, there was no evidence for reversal of rejection, and, unsurprisingly, in some instances, excessive antirejection therapy increased the frequency of infectious complications [9, 25]. Effective therapies to reverse AMR in XTx remain to be fully explored.

Based on the high frequency of AMR, the wide diversity of potential polymorphic porcine peptides and the chronic detection of vascular antibody deposition in GTKO donor hearts, it is necessary to establish methods for early diagnosis and effective treatment. It appears that anti-CD40-based immune suppression, which is likely to be used in clinical CXTx, relies heavily on effective costimulation blockade, as withdrawal of anti-CD40 therapy has resulted in the induction of non-Gal IgM and IgG [8]. This has at least two potential consequences. Firstly, costimulation blockade complicates the use of plasmapheresis, commonly used to treat AMR, as it would remove both therapeutic and pathogenic antibodies. Secondly, chronic dosing with biological therapeutics risks the development of anti-antibody immune responses [112, 113]. While the frequency of this response is difficult to estimate and cannot be safely extrapolated from the results reported for other antibody-based therapeutics, in the context of clinical CXTx, an anti-anti-CD40 response would be potentially serious. Few clinical methods can reverse antibody-mediated heart rejection so it will be important, prior to clinical CXTx, to develop and test, to as great a degree as possible, xenospecific therapies for detecting and treating AMR using a pig-to-NHP transplant model. This may include alternative versions of anti-CD40 (Table 2), alternative costimulation strategies [13], total lymphoid radiation [114], or current antibody reduction therapies [105, 106]. Ideally, such a study would be performed using CXTx, but life-supporting kidney XTx, with well-known physiological markers for organ function, maybe a more pragmatic solution as ongoing kidney rejection will not result in rapid recipient death. What is clear to investigators with experience in oCXTx is that the NHP model plays a critical role in progressing to clinical application, but has intrinsic limitations with particular regard to recipient management.

## 7. Infectious Disease Issues

Complete knowledge and risk-free application of clinical CXTx, as with most major advances in medicine, retains elements of uncertainty. The potential for disease transmission has been a significant concern for clinical XTx [115]. Concern has been expressed about the potential of porcine endogenous retroviruses (PERV) to emerge in XTx recipients, infect patient tissues, and adapt to humans [116]. Since this potential was identified, molecular and immunologic assays to monitor PERV infection have been developed [117], and significant advances have been made in mapping PERV proviral sites [118, 119] and understanding the basic biology of PERV infection [120]. Several clinical studies of patients exposed to porcine tissues [121–124] or in NHP XTx recipients [125] have also failed to detect PERV infection. The generation of high titer human-trophic PERV requires the recombination of relatively rare PERV-C proviral sequences with more common PERV-A. Selective breeding can be used to eliminate PERV-C from donor pigs [126]. Alternatively, nuclease-directed mutation of the PERV *pol* gene has been shown to induce widespread PERV proviral deletions [50], but this may be unnecessary if PERV-C is eliminated by selective breeding. While diligent monitoring of PERV infection in XTx recipients is prudent, the apparent risk presented by PERV appears to be small and is unlikely to delay clinical CXTx. Aside from endogenous retrovirus, specific pathogen-free (SPF) donor pig facilities have been produced and populated with caesarean-derived piglets. Some of these sites have been operational and breeding pigs for many years demonstrating the feasibility to routinely produce donor pigs with exceptionally high health standards.

## 8. Conclusion

It is clear that cardiac and renal XTx can benefit patients in need of organ replacement. If continued studies as outlined above are performed, we are optimistic that this technology will soon be ready for clinical testing. These remaining key preclinical studies are required to ensure the efficacy and safety of clinical CXTx. Principally a life-supporting preclinical oCXTx study in NHPs must be performed to demonstrate acceptable perioperative and postoperative recipient survival. This study should optimize organ preservation and utilize immune suppression based on anti-CD40 costimulation blockade. To meet ISHLT suggested standards [14], this study, involving at least 16 CXTx recipients, will require significant financial resources, an infrastructure to simultaneously maintain multiple CXTx postoperative recipients and a dedicated team of clinicians, veterinarians, scientists, and animal technologists. Donor organs should minimally contain GTKO;hCRP genetics, likely with additional antigen reduction of CMAH-KO and B4GALNT2-KO. Clinical use of additional genetic modifications should be founded on further rigorous preclinical testing in NHPs to demonstrate their utility, which, in the case of CMAH, will likely require testing in an immunologically appropriate CMAH-KO large animal transplant model. As well as achieving adequate perioperative and postoperative recipient survival, such a preclinical study should allow for organ rejection. This rejection study, while unlikely to fully predict the clinical immune response, will suggest essential xenospecific assays and therapies to reverse rejection which can be further refined during clinical CXTx.

Genetic engineering has significantly improved CXTx organ survival but ongoing creation of new genetics, in pursuit of the perfect donor, has the potential to delay clinical studies. We believe the initial clinical studies will rely primarily on known systemic immune suppression and genetics and that further optimization of donor genetics is best pursued in response to identified, researched clinical immune and physiological requirements.

Dependent on the results of these prospective preclinical oCXTx studies in NHPs, it appears to us that the era of clinical CXTx is approaching. The scientific path forward is demanding but well defined; however, the complexity of any clinical XTx program, including the heart, presents a substantial and unique set of regulatory challenges which need to be addressed expeditiously to avoid delaying the realization of clinical use.

## Figures and Tables

**Table 1:** The longest (median) reported heterotopic cardiac xenograft survival as a function of donor genetics and immune suppression.

Donor	Earlier immune suppression		Costimulation blockade	
	CsA/CyP/steroid	ATG/CD20/tacrolimus/sirolimus	ATG/LoCD2b/CVF/anti-CD154/MMF	ATG/anti-CD40, CD20/CVF/MMF
WT	32^§^ (21 d) [27] 25^‡^ (12 d) [28]	n.r.	n.r.	n.r.
WT;hCRP	99^Δ^ (26 d) [29] 78^∗^^Δ^ (35 d) [30]	109^∗^^#†^ (20 d) [31] 137^∗^^#†^ (96 d) [32]	139^∗^ (27 d) [36]	n.r.
GTKO	n.r.	128^†^ (22 d) [34]	179 (78 d) [37]	n.r.
GTKO;hCRP	n.r.	52^∇†^ (28 d) [34]	8^¶^ (8 d) 236^†¶^ (71 d) [5]	149^¶^ (84 d) [25]
GTKO;hCRP;TBM	n.r.	n.r.	n.r.	945^¶^ (298 d) [8]

n.r.: none reported. ^§^Soluble CR1 to block complement activation. ^‡^Cobra venom factor at 0.25–0.5 mg/kg prior to surgery and 0.1–0.5 mg/kg every 1–4 days thereafter. ^∗^Included use of alpha-Gal polymer GAS914 [127] or Nex1285 [128]. ^†^Immune suppression included anti-CD20 (Rituximab) B-cell depletion. ^Δ^hDAF (human CD55) minigene [129]. ^∇^A murine H-2Kb regulated human CD55 transgene [77]. ^#^hCD46 transgene based on 60 kb human genomic CD46 DNA [130]. ^¶^hCD46 transgene based on a human CD46 minigene [131].

**Table 2:** Anti-CD40 antibodies in clinical development.

Antibody	Company	Status	Trial ID
SGN-40	Seattle Genetics Inc.	Phase 1 multiple myeloma Phase 2 B-cell lymphoma Phase 1/2 chronic lymphocytic leukemia	NCT00079716 NCT00435916 NCT00283101

ASKP 1240	Astellas	Phase 2 renal Tx phase 2 plaque Psoriasis	NCT01780844 NCT01585233

HCD122	Novartis	Phase 1, chronic lymphocytic leukemia Phase 2 multiple myeloma Phase 2 follicular lymphoma Phase 1/2 Hodgkin's and non-Hodgkin's lymphoma	NCT00108108 NCT00231166 NCT01275209 NCT00670592

Chi Lob 7/4	Cancer Res UK	Phase 1 cancer malignancies	NCT01561911
BG9588	NIDDK	Phase 2 renal Tx	NCT00001857
	NIAMS	Phase 2 lupus nephritis	NCT00001789

## Abbreviations

AMR: Antibody-mediated rejection
B4GALNT2: Beta 1,4-N-acetylgalactosamine transferase-2
CC: Consumptive coagulopathy
CCS: Cyclophosphamide, cyclosporine, and steroids
CMAH: CMP-N-acetylneuraminic acid hydroxylase
CVF: Cobra venom factor
CXTx: Cardiac xenotransplantation
GTKO: Alpha-galactosyltransferase (GGTA_1) mutant
hCRPs: Human complement regulatory proteins
hCXTx: Heterotopic CXTx
ISHLT: International Society of Heart and Lung Transplantation
Neu5Gc: N-Glycolylneuraminic acid
NHP: Nonhuman primate
oCXTx: Orthotopic CXTx
PCXD: Perioperative cardiac xenograft dysfunction
PERV: Porcine endogenous retroviruses
RXTx: Renal xenotransplantation
SPF: Specific pathogen free
TBM: Thrombomodulin
WT: Gal-positive pig
XTx: Xenotransplantation.
